# Molecular Simulation of Electron Traps in Epoxy Resin/Graphene Oxide Nanocomposites

**DOI:** 10.3390/polym14194208

**Published:** 2022-10-07

**Authors:** Xuanning Zhang, Hao Xu, Yanyu Liang, Geng Chen, Shaocong Wu, Guohui Hao, Youping Tu, Cong Wang, Yongsheng Xu, Haoou Ruan

**Affiliations:** 1 State Key Laboratory of Alternate Electrical Power System with Renewable Energy Sources, the Beijing Key Laboratory of High Voltage and EMC, North China Electric Power University, Changping, Beijing 102206, China; 2 State Key Laboratory of HVDC, Electric Power Research Institute, China Southern Power Grid, Huangpu, Guangzhou 510080, China

**Keywords:** electron affinity energy, epoxy resin, nano graphene oxide, interface, electron trap

## Abstract

Trapped space charges in epoxy composite distort the electric field, which will induce the failure of the insulation system, and nano graphene oxide may inhibit the curing behavior of epoxy resin matrix. This paper analyzes how the two interfaces affect the electron traps of epoxy resin/graphene oxide systems with different nanofiller contents. The electron affinity energy of epoxy resin matrix and nano filler molecules in the epoxy resin/graphene oxide system is calculated based on quantum chemistry. It is found that nano graphene oxide has a strong electron affinity energy and is easier to capture electrons. Then the influence of the interface formed by the epoxy resin matrix and the nano graphene oxide on the electron transfer ability is calculated. The epoxy resin matrix contains the electron transfer ability of interfaces formed by nano graphene oxide and the molecular chain is different from that of unreacted molecules. The results can provide a reference for the modification of epoxy resin/graphene oxide nanocomposites.

## 1. Introduction

Epoxy resin (EP) is widely used in the insulation of electrical equipment and electronic devices. However, the distortion of the electric field induced by space charge injection caused by high field and temperature gradient aggravates the breakdown and electrical tree of insulation materials [1,2,3,4], which seriously threatens the safe and stable operation of power equipment. Therefore, it is urgent to study high-performance direct current insulation materials. Previous experimental results show that graphene oxide content is closely related to electron traps of epoxy resin/graphene oxide nanocomposites [5,6,7]. The doping of nanofillers in polymer materials can effectively improve their electron trap characteristics, thereby inhibiting the charge injection of high-voltage electrodes. At present, it is generally believed that the interface formed by nanofiller and polymer matrix is the key to the change of electron trap characteristics of nanocomposites. Japanese Tanaka et al. proposed the “multi-core” model of the interface region [8], which divides the nanofiller and matrix interface into three regions: bonded layer, bound layer, and loose layer, and the electric double layer overlapped on the above three layers. Li et al. synthesized the interface model of nanocomposites and divided the interface region into the bonded region and transition region [9]. The overlapping region of the transition region has less binding ability to carriers. On this basis, the barrier model of nanocomposite dielectric is proposed [10]. This model believes that the bonded region near the nanoparticles will form a higher barrier, which is the deep electron trap. The matrix near the transition region forms a lower barrier, and shallow traps gradually dominate. Therefore, it can be found that the interface between nanofiller and matrix directly affects the change of electron trap.

The charge transport process in insulating materials is often described by the energy band theory. The electrons in the valence band transmit to the conduction band through the forbidden band to form the electron transport in the material [11]. Some electrons are confined to a certain atom in the transition process, that is, electrons are captured by the localized state [12]. The common localized states include polar groups, impurities, molecular defects, etc. The electron traps in the materials improve the electric field distortion caused by charge accumulation and ultimately improve the insulation strength of materials. Nano graphene oxide (GO) is a graphene oxide nanosheet composed of sp^2^ hybrid carbon atoms, which has high surface energy and large specific surface area and can effectively improve the thermal stability and mechanical properties of polymer materials [13,14,15]. Nelson et al. found that a nano graphene oxide volume fraction of 3 vol.% and 5 vol.% composite material has excellent nonlinear conductivity characteristics [16]. Du et al. found that when there is the introduction of an appropriate amount of nano graphene oxide into low-density polyethylene materials, it will have more deep traps, thereby inhibiting the accumulation of space charge and the distortion of the electric field, but when the doping nanofiller content is high, the trap level becomes shallow [15,17]. Liu et al. studied the characteristics of single-layer and multi-layer graphene oxide doped epoxy composites and found that EP/GO composites not only inhibit charge transfer but also introduce deep traps [18]. It can be found that studies have shown EP/GO as the insulator material in gas insulation equipment has a good prospect. It not only has excellent mechanical and thermodynamic properties but also can improve the dielectric properties of the material by regulating the trap characteristics. However, when the doping nanofiller content is high, it is not conducive to improving the material properties.

Quantum chemistry can be used to calculate microproperties such as band structure, molecular orbitals, and electrostatic potential distribution based on microscopic models. It is used to solve the Schrödinger equation based on several approximations using methods which include the Hartree–Fock self-consistent method and DFT. We can gain insight into the mechanism of space charge behavior and trapping processes using the quantum chemistry calculation. Lahaye et al. found that graphene oxide with a high oxidation degree has a wider band gap by density functional method and is easier to capture electrons [19]. Meunier et al. studied the defects on the molecular chain of decane and the influence of impurities on the electron residence time and electron trap level, and through calculation, found that the electron traps’ level of different groups is different. The electron trap level introduced by hydroxyl and non-conjugated C=C double bonds is shallower, while the carbonyl and conjugated double bonds are easier to introduce deep electron traps [20,21]. Li et al. studied the effects of different electric fields and temperatures on the electron traps of polymer based on molecular dynamics [22]. Li et al. studied the mechanism of trap characteristics change of EP/fullerene composites by the quantum chemistry method. They found that fullerene has high electron affinity and formed more capture sites in the material, which played a role in inhibiting space charge accumulation [23]. However, they only considered the influence of a single molecule on electron traps, ignoring the influence of the interface between the nanofiller and the matrix.

In this paper, the influence of the interface between epoxy matrix and nano graphene oxide filler on trap distribution is studied through the analysis of the density of states, electron affinity, and electron capture energy. The microscopic mechanism of the influence of the interface on trap distribution is revealed.

## 2. Simulation and Experimental Methods

In this section, the calculation methods of electron affinity energy and electron transfer are described. Afterwards, the electron capture energy of different polar groups on the nano graphene oxide are calculated according to the electron affinity energy. Finally, the preparation of EP/GO sample and the experimental method of electron traps are introduced.

### 2.1. Calculation Method of Electron Capture and Transfer Ability in EP/GO

**Figure 1:**
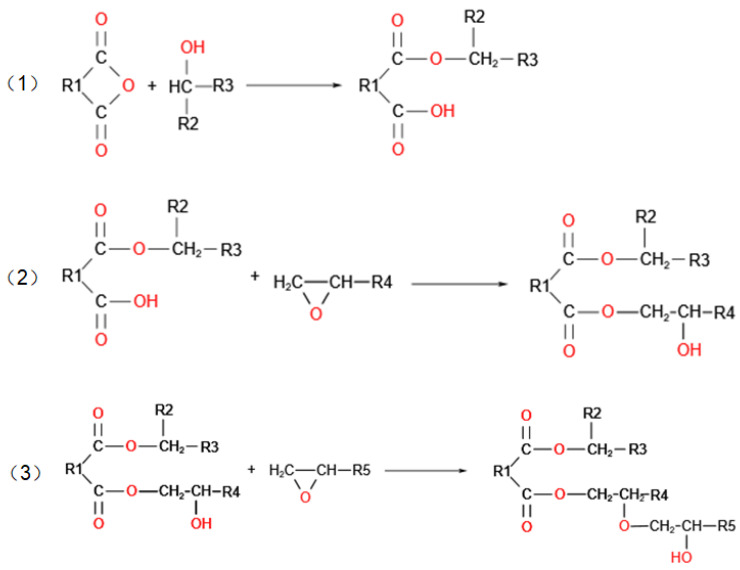
Curing reaction mechanism of epoxy resin: (1) the anhydrides are ring-opened and react with hydroxyl groups in epoxy resin to form monoesters containing carboxylic acids; (2) the carboxylic acid reacts with the epoxy group to form new hydroxyl groups; (3) the hydroxyl generated in the previous step reacts with the epoxy group to generate new hydroxyl, and the generated hydroxyl also further reacts with the anhydride group to generate carboxylic acid.

The EP/GO system contains nano graphene oxide, epoxy resin molecular chains, and unreacted molecules (including the epoxy resin and curing agent molecules). The epoxy resin molecular chain is modeled according to the curing reaction that is shown in Figure 1. To characterize the attraction between different molecules and electrons, the electron affinity energy (*EA*) is calculated based on density functional theory (DFT) [24]. The *EA* of the molecule describes the change of energy when the molecule obtains an electron, which is the energy difference formed by the electron from the vacuum level to the conduction band level. The *EA* is described in [21]:

(1) $$EA=E(R_{e})-E(R_{e}^{-1})$$

where the *E*(R_e_) and the *E*(R_e_^−1^) is the total energy of neutral molecule and anion with one electron in equilibrium geometry, respectively.

Meanwhile, nano graphene oxide molecule is connected with abundant oxygen-containing functional groups. Due to the different abilities of different atoms to attract electrons, the electronic orbital around the atom is biased, resulting in polar groups capturing electrons. The *EA* of graphene and graphene oxide is calculated according to Equation (1). The *EA* of these nanofillers is positive, and the attraction to electrons is stronger. Therefore, the nano graphene oxide can capture electrons in the composites, and the oxygen-containing functional groups on graphene oxide can also improve the *EA* of molecules. The electron capture energy of different groups of nano graphene oxide can be described in [21]:

(2) $$E_{Capture}^{}=EA^{defect}-EA^{origin}.$$

where the *E_Capture_* is the electron capture energy, the *EA^defect^* and *EA^origin^* are the *EA* of graphene oxide and graphene molecules, respectively.

The electron exchange-correlation interactions were expressed with a generalized gradient approximation (GGA) in the form of the Perdew–Burke–Ernzerhof (PBE) functional. The GGA-PBE functional has been successfully used to describe the interaction between organic molecules and a carbon-based substrate or an inorganic substrate [25]. In addition, there are different kinds of common functional, including Perdew-Wang 91 (PW91) and Becke3-Lee-Yang-Parr (B3LYP). The construction of PBE and PW91 is similar, however, PBE’s effective potential is smoother and prone to numerical stabilities [26]. B3LYP is not suitable for large conjugate systems [27]. Integration over the Brillouin zone was performed with 2 × 1 × 2 *k*-points. The self-consistent field tolerance is 1 × 10^−6^ Hartree. The double numerical plus polarization (DNP) was selected and the DFT semi-core pseudopotential (DSPP) was applied for core treatment. The DNP basis set is important for hydrogen bonding which is suitable for calculating the model containing polar groups. In addition, the interactions related to dispersion terms are taken into account by choosing the Grimme method [28]. 

### 2.2. EP/GO Sample Preparation and Isothermal Surface Potential Decay Method

EP/GO composites doped with 0 wt%, 3 wt%, and 5 wt% nano graphene oxide are prepared. Epoxy resin is E51 bisphenol-A epoxy resin (DGEBA), the curing agent is MeTHPA, and graphene oxide is produced by Ang Xing Company. The preparation process of the composite is described as, at first, 0.01 kg epoxy resin and the corresponding nanofiller content of graphene oxide and tetrahydrofuran mixed solution are added into a 0.5 L beaker; stirring evenly and ultrasonic dispersion for 10 min; steam tetrahydrofuran in an oven; then 3.8 × 10^−3^ kg curing agent is added and degassed at 333.15 K for 1 h in the vacuum oven; finally, the composite samples with the above nanofiller content are prepared by vacuum casting and curing at 403.15 K for 28 h.

**Figure 2:**
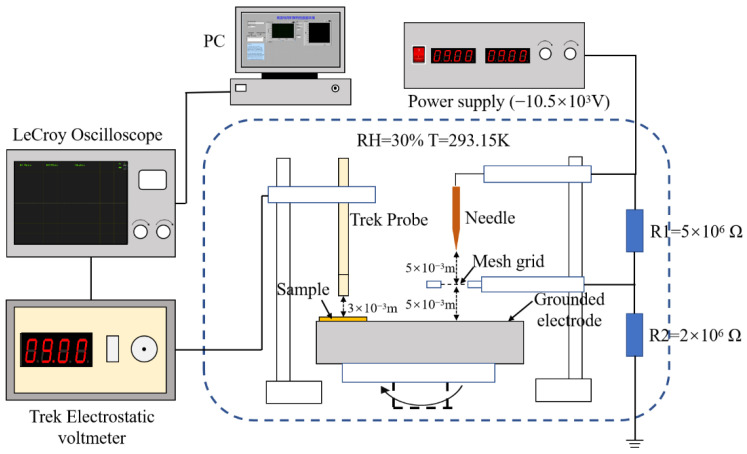
Structure of ISPD experimental equipment.

The isothermal surface potential decay (ISPD) method is used to test the electron trap distribution spectrum of the prepared samples. ISPD is a method to study the electron traps of materials by measuring the change of surface potential. Under the action of the electric field, the electrons generated by corona near the high voltage electrode migrate evenly to the surface of the sample through the mesh grid, then measure the surface potential change of the sample using a probe. The structure of the experimental equipment is shown in Figure 2.

## 3. Results and Discussion

In this section, the electron affinity energy of different molecules in the EP/GO system is calculated using the calculation method in Section 2, and calculate the electron capture energy of different polar groups of nano graphene oxide. Afterwards, the electron transfer energy barrier of the interface between epoxy resin matrix and nano graphene oxide analyzes by the density of states. Then, the electron traps of epoxy composites with nanofiller content of 0, 3, and 5 wt% are tested by the ISPD method. Finally, the correlation between simulation and experimental results is discussed.

### 3.1. Results

#### 3.1.1. Analysis of Electron Affinity Energy in EP/GO

Epoxy resin is the polymer formed by the curing reaction of epoxy resin molecules and curing agent molecules. As nano graphene oxide will inhibit the curing reaction and produce more unreacted molecules [29,30], this paper mainly considers the effect of unreacted molecular defects on the electron traps.

**Figure 3:**
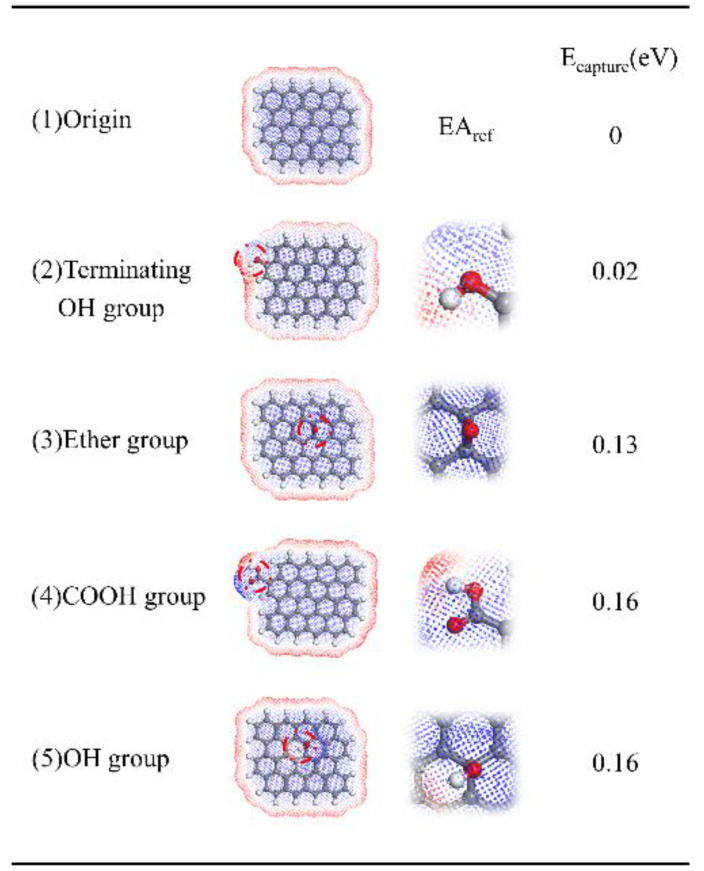
The electron capture energy of different groups in the nano graphene oxide: (1) the electron capture energy of the original molecule; (2) the electron capture energy of the terminating OH group in the nano graphene oxide; (3) the electron capture energy of the ether group in the nano graphene oxide; (4) the electron capture energy of the COOH group in the nano graphene oxide; (5) the electron capture energy of the OH group in the nano graphene oxide.

The *EA* of different molecules can be found in Table 1; the *EA* of DGEBA and curing agent molecules is negative, difficult to attract electrons, and the *EA* of epoxy resin molecular chain is high, easier to attract electrons. The electron capture energy can be found in Figure 3. The positive *E_capture_* indicates that the *EA* of the defect molecule is higher than that of the original molecule, and the electrons in the conduction band will leave and be captured by the defect, reducing the total energy. It was found that the oxygen-containing functional groups on the nano graphene oxide have a large electron capture energy, which contributes to the electron trap, and the *EA* of nano graphene oxide is higher than the epoxy resin molecular chain and the unreacted molecule. Nano graphene oxide plays the role of capturing electrons in composite materials.

#### 3.1.2. Analysis of Interface Energy Barrier in EP/GO

The epoxy resin molecular chain and DGEBA molecules are insulating materials, so there is a forbidden band between the conduction band and the valence band, which electrons are difficult to transfer. Next, we analyze the electron transfer ability of the interface between epoxy resin matrix and nano graphene oxide is analyzed.

**Figure 4:**
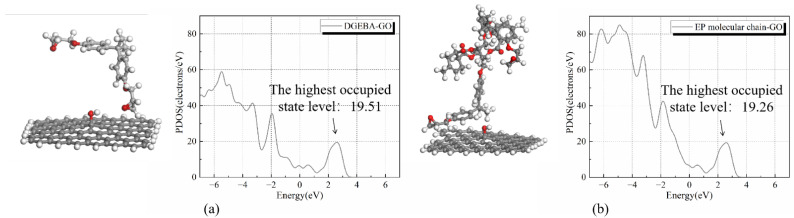
The PDOS distribution of interfaces in EP/GO: (**a**) interface 1-DGEBA and GO; (**b**) interface 2-EP molecular chain and GO.

Figure 4 depicts the two kinds of interface and their PDOS, which are interfaces formed by GO with DGEBA and EP molecular chain. Through the comparison of Figure 4a,b, the highest occupied state level (HOSL) formed by the interface between the epoxy resin molecular chain and the nano graphene oxide is lower, which is 19.26 electrons/eV. Because the energy for electrons to enter the molecular orbital is lower, the electrons are prone to enter this kind of interface and be captured by the electron traps.

**Figure 5:**
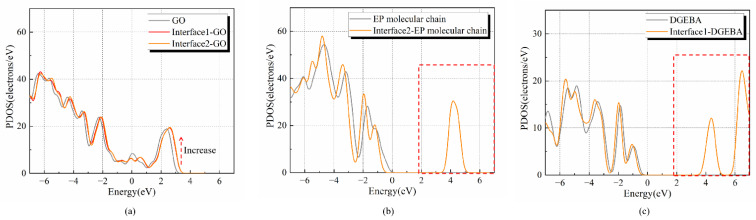
Comparison of the PDOS distribution between EP and GO molecules in interfaces: (**a**) PDOS distribution of GO alone and GO at the interfaces; (**b**) PDOS distribution of the EP molecular chain alone and molecular chain at the interface; (**c**) PDOS distribution of DGEBA alone and DGEBA at the interface. (The red dotted line part is the number of electrons in molecular orbitals with high energy.)

Figure 5 shows that the *EA* of nano graphene oxide is higher than that of the epoxy resin molecular chain and DGEBA molecule. After forming the interface, the amount of electrons in the molecular orbitals with high energy of epoxy resin molecular chains and DGEBA molecules becomes smaller, which can be found in the red dotted line part in Figure 5b,c. Meanwhile, the HOSL of DGEBA and GO interface becomes higher. Due to its stronger *EA*, nano graphene oxide attracts electrons more easily from the epoxy resin matrix.

#### 3.1.3. Electron Traps Analysis of EP/GO Material

**Figure 6:**
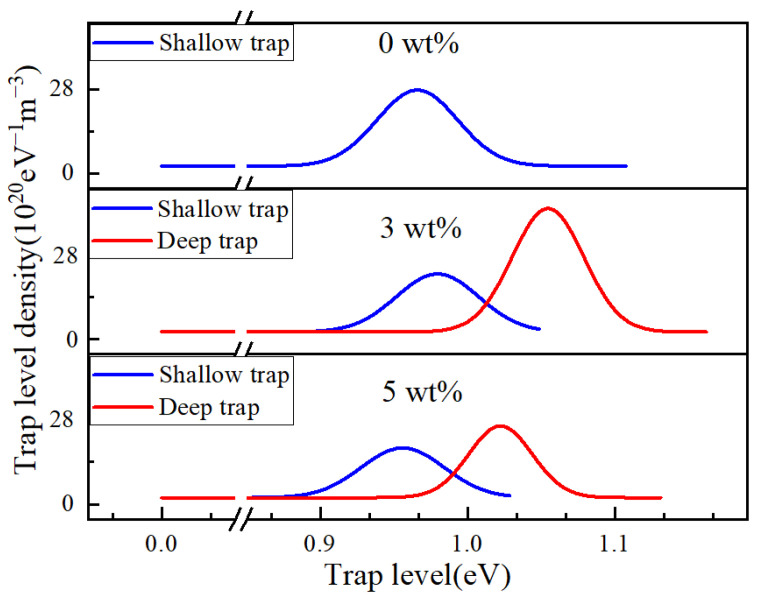
The electron traps distribution spectrum of different nanofiller contents of EP/GO.

Simmons theory is used to analyze the surface potential decay curve [31], and the electron trap distribution spectra of EP/GO with different nanofiller contents are obtained in Figure 6.

Figure 6 shows that there is only one peak in pure epoxy resin; the corresponding electron trap energy level is 0.96 eV, and the electron trap level density is 2.76 × 10^21^ eV^−1^m^−3^. EP/GO composites have two peaks, which are defined as the shallow electron trap and deep electron trap. Shallow and deep electron traps are defined according to the relative value of the electron trap level. The bigger one is a deep electron trap and the smaller one is a shallow electron trap. In the composite, the electrons in the shallow electron traps are easier to release, however, the electrons in the deep electron traps are involved in the electron-release process as time increases. The electron trap level of 3 wt% composite becomes deeper, and the shallow and deep electron trap levels are 0.98 and 1.05 eV, respectively. The electron trap level density is 2.15 × 10^21^ and 4.32 × 10^21^ eV^−1^m^−3^, respectively. The shallow and deep electron trap levels of 5 wt% composites are 0.95 and 1.02 eV, and the electron trap level density is 1.87 × 10^21^ and 2.59 × 10^21^ eV^−1^m^−3^, respectively.

### 3.2. Discussion

Due to nano graphene oxide affects the curing behavior of epoxy resin matrix, nano graphene oxide covers the reaction sites and curing agent molecules in epoxy resin molecules because of its high surface area, so nano graphene oxide will hinder the curing reaction of epoxy resin matrix. Therefore, in the EP/GO with high nanofiller content, more nano graphene oxide has a stronger effect on inhibiting the curing reaction of the epoxy resin matrix, and there will be more unreacted molecules. By comparing the two kinds of interface models of epoxy resin molecular chain and nano graphene oxide, unreacted molecule and nano graphene oxide, the reasons for the change of electron traps in EP/GO system with different nanofiller contents are studied.

In the 3 wt% EP/GO composite system, the nano graphene oxide has a weak inhibitory effect on the curing behavior of the epoxy resin matrix, there are fewer unreacted molecules, and the interface between the epoxy resin molecular chain and the nano graphene oxide is dominant. The HOSL of the interface formed by epoxy resin molecular chain and nano graphene oxide is low, the energy barrier that the electron transfer needs to overcome decreases, and the electron transfer is easy to enter the molecular orbitals of the interface. Meanwhile, the epoxy resin molecular chain has a large *EA* and is easier to attract electrons, so the electron trap level increases. In the 5 wt% EP/GO composite system, nano graphene oxide further inhibited the curing behavior of the epoxy resin matrix, and the interface between the unreacted molecules and nano graphene oxide is dominant. The HOSL of the interface between the unreacted molecule and the nano graphene oxide is big, and the energy barrier that the electron transfer needs to overcome increases. The *EA* of the unreacted molecule is relatively low, the ability to attract electrons is weak, and the electron trap level and deep electron trap level density become smaller.

**Figure 7:**
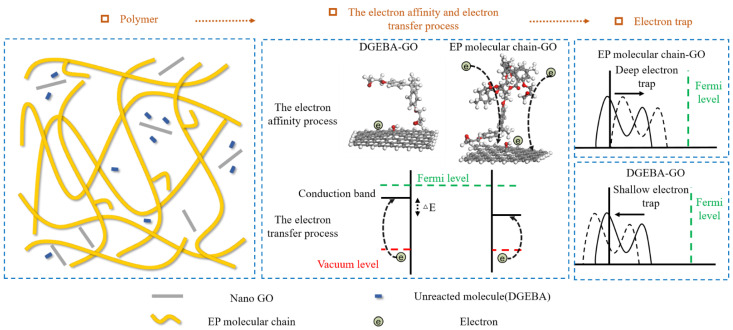
Analysis of electron traps in EP/GO nanocomposites.

Summing up, the electron trap of nanocomposites is affected by the interface formed by nano graphene oxide and epoxy resin matrix. The EP/GO system with different nanofiller contents have different inhibitory effects on the curing behavior of the epoxy resin matrix, and the dominant interface in nanocomposites is also different. When the interface between epoxy resin molecular chain and nano graphene oxide is dominant, the electrons are easy to be trapped, and the electron trap level is high. When the interface between unreacted molecules and nano graphene oxide is dominant, the electrons are difficult to be trapped, and the electron trap level and deep electron trap level density are low. The analysis process is shown in Figure 7. Therefore, with the increase of nanofiller content in EP/GO, the electron trap level increases first and then decreases.

## 4. Conclusions

In summary, we analyze the effects of two kinds of interface formed by epoxy resin and nano graphene oxide on electron capture and transfer ability. The reasons for the change of electron traps in nanocomposites with different nanofiller contents is discussed. In the 3 wt% EP/GO system, although nano graphene oxide can inhibit the curing behavior of epoxy resin matrix, the electron trap level becomes deeper due to the strong *EA* of nano graphene oxide and the interface of nano graphene oxide with epoxy resin molecular chains. In the 5 wt% EP/GO system, nano graphene oxide significantly inhibits the curing behavior of epoxy resin matrix, increases the number of unreacted molecules, reduced the ability to capture electrons, and the energy barrier which electrons need to overcome is high, and the electron trap level becomes shallow. Generally, this interesting phenomenon presented in this paper has physical implications in revealing relations between the epoxy resin matrix and the nano graphene oxide interface and electron traps.

## Figures and Tables

**Table 1:** *EA* of different molecules.

Molecules	*EA* (eV)
Curing agent	−0.387
DGEBA	−0.781
Epoxy resin molecular chain	0.013
Graphene oxide	0.073–0.079
Graphene	0.073

## Data Availability

The data presented in this study are available on request from the corresponding author.
